# Complete atrio-ventricular block with coronary artery spasm due to direct laryngoscopy in a pediatric patient with laryngeal papillomatosis: a case report

**DOI:** 10.1186/s40981-021-00437-9

**Published:** 2021-04-17

**Authors:** Katsuyuki Matsushita, Risa Arai, Tetsuzo Nakayama, Toshiaki Nakagaki, Tadashi Kandabashi, Ken Yamaura

**Affiliations:** 1 Operating Rooms, Kyushu University Hospital, 3-1-1 Maidashi, Higashi-ku, Fukuoka, 812-8582 Japan; 2 Department of Anesthesiology and Critical Care Medicine, Kyushu University Hospital, Fukuoka, Japan; 3 Medical Information Center, Kyushu University Hospital, Fukuoka, Japan; 4 Department of Anesthesiology and Critical Care Medicine, Graduate School of Medical Sciences, Kyushu University, Fukuoka, Japan

**Keywords:** Coronary artery spasm, Direct laryngoscopy, Atrio-ventricular block, Laryngeal Papillomatosis

## Abstract

**Background:**

Coronary artery spasm has rarely been reported in pediatric patients. Previous studies have reported comorbidities and risk factors for coronary artery spasms. We present the case of a complete atrio-ventricular (AV) block that occurred in the absence of other risk factors immediately after direct laryngoscopy.

**Case presentation:**

A 2-year-old girl developed severe coronary artery spasm after direct laryngoscopy for elective laryngeal papillomatosis resection. Immediately after the initiation of laryngoscopy, complete AV block and ST elevation on lead II of the electrocardiogram were observed. These findings indicated that the complete AV block was caused by a right coronary artery spasm.

**Conclusion:**

Coronary artery spasm resulting in lethal arrhythmia rarely occurs in healthy pediatric patients. To the best of our knowledge, this is the first pediatric case of a severe coronary artery spasm resulting in a complete AV block due to direct laryngoscopy in a healthy patient.

## Background

Coronary artery spasm has rarely been reported in pediatric patients [1–5]. Patients with a history of coronary artery spasm have comorbidities such as Kawasaki disease [4], systemic lupus erythematosus [5], and heart disease [2, 3]. A previous review [6] revealed that perioperative coronary spasm occurred in men with associated risk factors; however, none of the patients were under 30 years of age. Vascular smooth muscle hyper-reactivity and endothelial dysfunction have been described as the main etiologies of coronary artery spasms. Furthermore, the role of an imbalance between parasympathetic and sympathetic tone in triggering coronary artery spasm is well known [7]. Due to an immature autonomic nervous system, children often experience hypervagotonia. In the perioperative period, nasopharyngeal or esophageal stimulation, such as nasogastric or endotracheal tube placement, often stimulates the vagus nerve; however, bradycardia resulting from such stimulation is usually transient and rarely turns severe. Herein, we report a pediatric patient without comorbidities who developed a complete atrio-ventricular (AV) block with right coronary artery spasm induced by direct laryngoscopy. Written informed consent was obtained from the parents of the patient for the publication of this report. This manuscript adheres to the CARE guidelines.

## Case presentation

**Fig. 1:**
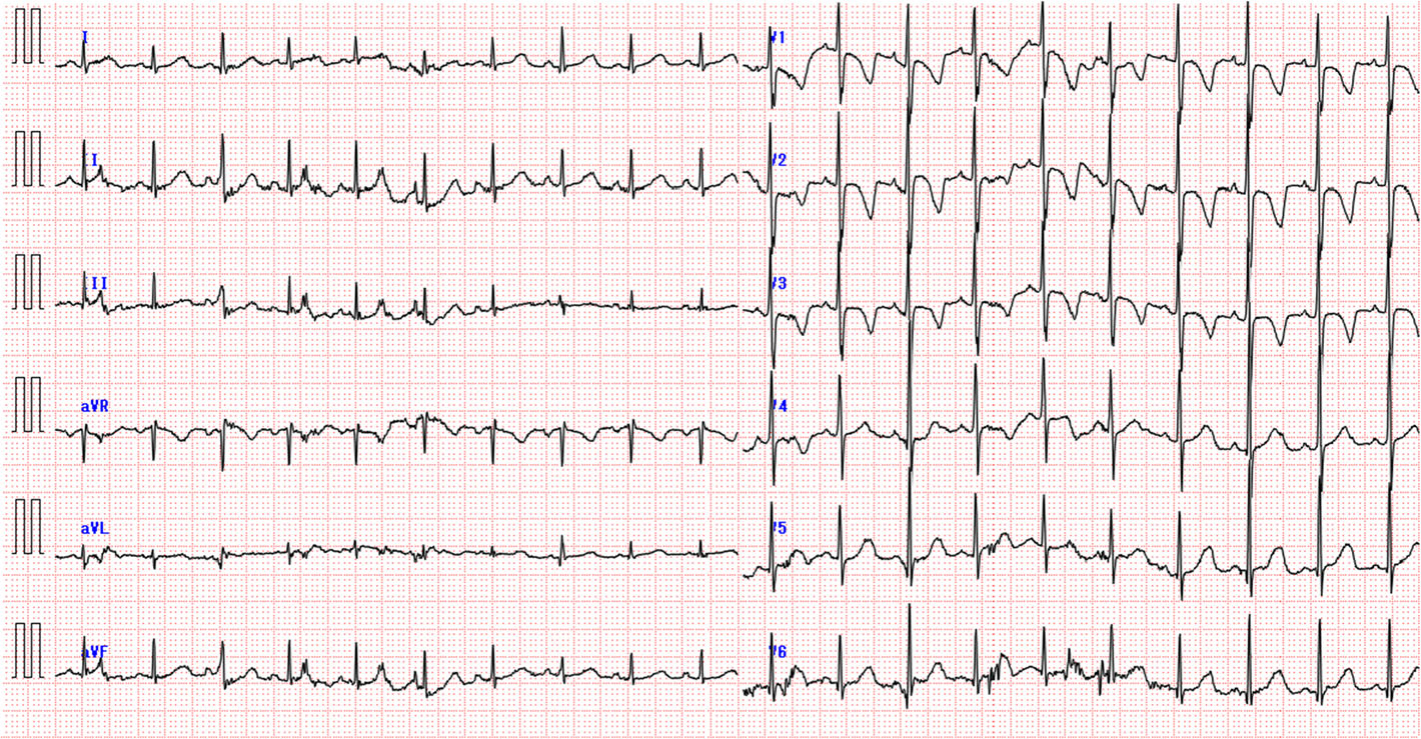
Pre-operative 12-lead electrocardiogram revealing normal sinus rhythm with normal QRS complexes and ST segments

A 2-year-old girl (height, 91.3 cm; weight, 12.2 kg) presented to our hospital with hoarseness of voice, which began soon after the patient started speaking. Dyspnea, laryngeal stridor, and snoring were absent. Laryngeal papillomatosis was diagnosed, and the patient was scheduled for a resection of the lesion. The patient did not have any significant medical history, and preoperative examinations, including chest radiography and electrocardiogram (ECG), were normal (Fig. 1). The preoperative blood pressure was 102/58 mmHg, and the heart rate was 110 beats/minute.

**Fig. 2:**
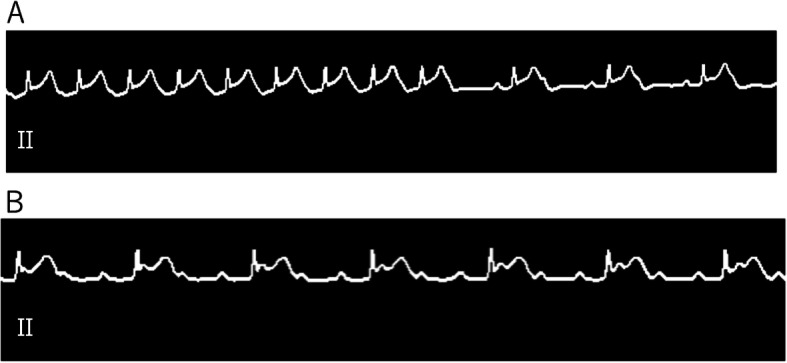
Electrocardiogram monitor showing ST-segment elevation and bradycardia after initiation of laryngoscopy (**a**). Complete atrio-ventricular block with ST-segment elevation is observed (P waves with a regular P-to-P interval, QRS complexes with a regular R-to-R interval, and variable PR interval) on the electrocardiogram monitor (**b**)

Inhalational anesthesia was induced with 5% sevoflurane and 50% nitrous oxide in oxygen. After the intravenous administration of suxamethonium, tracheal intubation was performed with an 8.0-Fr airway exchange catheter (AEC) for jet ventilation during surgery. Anesthesia was maintained with continuous infusion of remifentanil (0.3 μg kg^−1^ min^−1^) and propofol (10 mg kg^−1^ h^−1^). A bolus dose of rocuronium was administered as required. Arterial pressure and bispectral index (BIS®) monitors were used in addition to standard monitors. Jet ventilation via AEC was adjusted on the basis of the arterial blood gas readings. The rate of remifentanil infusion was increased to 0.5 μg kg^−1^ min^−1^ 2 min before the surgery, and direct laryngoscopy for laryngeal papillomatosis resection was initiated by the surgeon. Immediately after the larynx was exposed with a laryngoscope, the patient’s heart rate increased from 77 beats/minute to 101 beats/minute. After 1 min, a complete AV block with ST segment elevation on lead II of the ECG and reduction in the arterial blood pressure from 128/84 mmHg to 49/35 mmHg occurred. The minimum heart rate was 47 beats/minute (Fig. 2). Laryngoscopy was aborted, and remifentanil infusion was stopped. Atropine (0.5 mg) was administered, and a continuous infusion of trinitroglycerin (0.5 μg kg^−1^ min^−1^) was initiated. The complete AV block and ST segment elevation were reversed 14 min later. Right coronary artery spasm due to direct laryngoscopy was suspected; therefore, trinitroglycerin infusion was continued, and remifentanil infusion was restarted at an increased rate (0.6 μg kg^−1^ min^−1^). Under these conditions, laryngoscopy was attempted a second time. No ECG or hemodynamic abnormalities were observed, and the surgery was completed without further problems. No abnormalities were observed during the postoperative echocardiographic examination, and the postoperative ECG was the same as the preoperative ECG. The patient was discharged 2 days after the surgery.

## Discussion

Coronary artery spasm is defined as intense vasoconstriction of the coronary arteries that causes total or subtotal vessel occlusion. It plays an important role in myocardial ischemic syndromes, including stable and unstable angina, acute myocardial infarction, and sudden cardiac death [8]. The characteristics of coronary artery spasm are that it occurs at rest and is not provoked by exertion, is associated with ST segment elevation on the ECG, and is often more prolonged than ischemic angina pectoris [9]. The mechanisms underlying coronary artery spasm remain unexplained; however, autonomic nervous system imbalance and endothelial dysfunction are known to be significant factors.

Coronary artery spasm is recognized as one of the causes of sudden death in adults; however, it is rarely observed in pediatric and adolescent patients in the absence of confounding risk factors [10]. Few cases of coronary artery spasm have been reported in pediatric patients. Subramanian et al. reported that a 15-month-old girl without coronary aneurysm developed acute myocardial infarction 3 months after treatment for Kawasaki disease [4]. Jasmin et al. reported that a 13-year-old girl with systemic lupus erythematosus experienced left-sided chest pain, ECG changes, and troponin elevation, which were suggestive of acute myocardial infarction, possibly due to coronary artery spasm. In these cases, the patients had comorbidities and risk factors related to endothelial dysfunction or small vessel disease, which may have led to coronary artery spasm. In our case, a complete AV block occurred in the absence of other risk factors after a direct laryngoscopy was performed by the surgeon. Simultaneously, ST elevation in lead II of the ECG was observed. These clinical findings suggest that vagal stimulation due to direct laryngoscopy resulted in right coronary artery spasm, which decreased the blood flow to the AV node and caused a complete AV block. The branches of the vagus nerve are distributed in the pharyngeal and laryngeal mucosa. Mechanical stimulation by the laryngoscope or tracheal tube stimulates the vagal cardiac branch through the medullary vagal nucleus, which induces bradycardia [11].

Moreover, propofol and remifentanil induce bradycardia, which has an inhibitory effect on the sympathetic nervous system [12]. In the present case, vagal stimulation by direct laryngoscopy and the use of propofol and remifentanil led to an imbalance between the parasympathetic and sympathetic nervous systems, which may have caused severe right coronary artery spasm and the complete AV block. We believe that the infusion rates of remifentanil and propofol were sufficient for direct laryngoscopy; however, pediatric patients might need more profound anesthesia than adults, and we should have been more careful while performing direct laryngoscopy.

Pediatric life support algorithm suggests the consideration of atropine administration for bradycardia caused by an increased vagal tone [13]. However, we should have considered epinephrine administration or the use of an external pacemaker because atropine, which acts at the AV node, is rarely effective in patients with a complete AV block. Continuous infusion of trinitroglycerin might have worked prophylactically against coronary artery spasm during the second laryngoscopy.

We searched the PubMed database on 27 March 2021 using the following keywords: <coronary artery spasm or coronary vasospasm> AND <child> or <coronary artery spasm or coronary vasospasm> AND <complete atrioventricular block>. A total of 109 papers and 38 papers were found respectively, but reports describing coronary artery spasms secondary to direct laryngoscopy in healthy pediatric patients were not found. We additionally checked the references cited in these papers and found no reports on pediatric patients suffering from a coronary artery spasm following a direct laryngoscopy. Therefore, to the best of our knowledge, this is the first report of a severe coronary artery spasm in a healthy pediatric patient at the time of direct laryngoscopy.

## Data Availability

Not applicable.

## Abbreviations

AV: Atrio-ventricular
ECG: Electrocardiogram
AEC: Airway exchange catheter
BIS: Bispectral index
